# Conjugated microporous polymer foams with excellent thermal insulation performance in a humid environment†

**DOI:** 10.1039/d1ra01616d

**Published:** 2021-04-14

**Authors:** Nianyun Feng, Shujuan Wu, Danna Song, Yimeng Li, Naijia Lu, Lei Sun, Tie Yu, An Li, Weiqiao Deng

**Affiliations:** Institute of Frontier and Interdisciplinary Science, Shandong University Qingdao 266200 China yutie@sdu.edu.cn; College of Petrochemical Technology, Lanzhou University of Technology Langongping Road 287 Lanzhou 730050 China lian2010@lut.cn

## Abstract

This work reported two monolithic conjugated microporous polymer (CMP) foams synthesized through the Sonogashira–Hagihara cross-coupling reaction without mechanical stirring. The as-synthesized (CMP-ED and CMP-PT) foams exhibited superior hydrophobicity and low apparent density of 58 mg cm^−3^ and 63 mg cm^−3^. In addition, CMP-ED displayed a low thermal conductivity of 34.04 mW m^−1^ K^−1^, which was comparable with commercial SiO_2_ aerogels (34.09 mW m^−1^ K^−1^) at 50% humidity conditions. When the environment humidity was raised from 50% to 70%, the thermal conductivity of CMP-ED and commercial SiO_2_ aerogels improved by 0.12% and 7%, respectively. Furthermore, XRD, FTIR, BET and TG were conducted to evaluate the bulk structure and stability of CMP-ED and CMP-PT. The results illustrated the thermal conductivity values were greatly affected by the pore structure of foams. And the strong hydrophobicity and the narrow pore structure were responsible for the good thermal insulation performance under humid conditions. Considering the low density, superhydrophobicity, excellent physicochemical stability and impervious thermal conductivity in a high humidity environment, this CMP-ED presented great potential as an insulating material in a humid environment.

## Abstract 2

This work reported two monolithic conjugated microporous polymer (CMP) foams synthesized through the Sonogashira–Hagihara cross-coupling reaction without mechanical stirring.

## Graphical abstract

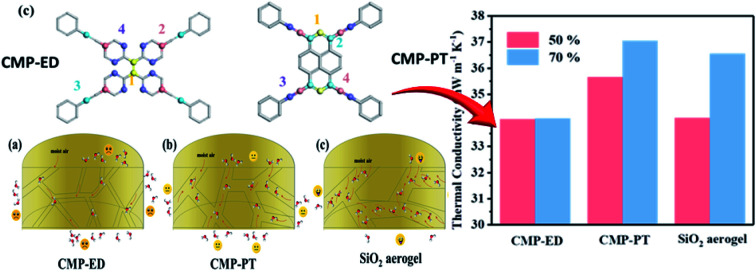

## 1 Introduction

Nowadays, thermal insulation materials are widely used in all aspects of our life, such as building materials, cold storage, and air conditioning equipment, and even stuffing in clothes, *etc.*^1^ Thermal conductivity is one of the main indicators to present the performance of thermal insulation materials (thermal conductivity is below 120 mW m^−1^ K^−1^), which depends on the composition, internal structure, bulk density, as well as the average temperature and humidity of the application environment, *etc.*^2–4^ Zhang and coworkers found that the porous structure could cause multiple reflections, which enhanced the insulation performance of the material.^5^ However, most of the porous insulation materials are susceptible to moisture, and their thermal conductivity not only increases greatly in humid conditions but also their mechanical properties decrease accordingly. Also, when the moisture content is above 5–10%, the steam diffusion and the movement of water molecules in the pores structure will greatly enhance the heat transfer, inducing insulation failure. Since the thermal conductivity of water is about 20 times greater than that of air, improving the hydrophobicity of porous materials could maintain the thermal conductivity under humid conditions. Therefore, it is necessary to explore a new type of heat insulation materials with low density, superhydrophobicity, low thermal conductivity rate and suitable aperture size.

Microporous organic polymers (MOPs) including intrinsic micro-porosity (PIMs), covalent organic frameworks (COFs), conjugated microporous polymers (CMPs), covalent triazine-based frameworks (CTFs) and hyper-crosslinked polymer (HCPs), and other nanomaterials, have been greatly developed in recent years due to their abundant ligand sources, unique skeleton structure, high specific surface area, and complex topological structure, *etc.*^2,3,6,7^ Up to now, MOPs have been widely used in many fields, such as supercapacitor,^8,9^ electrocatalysis,^10–12^ photocatalysis,^13,14^ photodegradation,^15^ lithium battery,^16^ solar steam generation,^17^ gas absorption and separation,^18^ CO_2_ capture and conversion^19,20^ and so on.

Especially, CMPs, firstly reported by Cooper^21^ with a large Π-conjugated network, rigid structure, and physicochemical stability, have attracted much attention in water treatment,^22–24^ supercapacitor^25,26^ electrocatalysis,^27^ and so on. Meanwhile, CMPs could generate a monolithic-foam with low density, superhydrophobic characteristics and certain compression resistance by own polymerization.^28–33^ Generally speaking, the wide application of CMPs materials is determined by its variety of Π-units, including simple phenyl units, extend aromatic hydrocarbon units and macro-rings. The unrestricted geometry enables CMPs to adjust skeleton structure flexibly and achieve the optimization between structure and performance after molecular structure modification.^34^ Therefore, it is meaningful to evaluate the thermal insulation performance of CMP materials in the humid environment in comparison to the commercial SiO_2_ aerogels.

Herein, we selected 1,1,2,2-tetrakis (4-bromophenyl) ethene and 1,3,6,8-tetrabromo-pyrene which possessed planar constructions and rich aromatic rings as ligands to synthesize the CMP foams. The two ligands may generate the CMP foams with superior hydrophobicity and micro-pore structure, which could adjust their heat insulation performance. The pore size of the CMP could be accurately controlled by the functional group structure of building units. Meanwhile, the bulk structure and the thermal stability were also evaluated. After the hydrophobicity test and the insulation performance measurement, we concluded the insulation potential of CMP foams and proposed the insulation mechanism behind the behavior.

## 2 Experimental

**Scheme 1:**
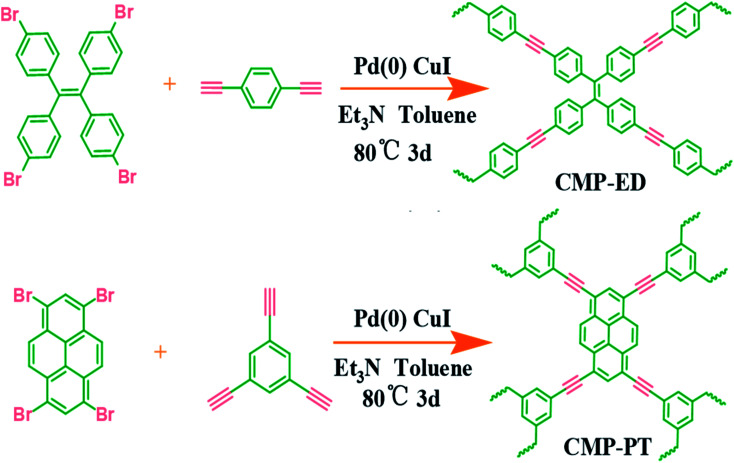
The synthetic routes of the CMP foams.

The synthesis method was according to our previous literature reports.^17^ The detailed routes of the CMP foams were shown in Scheme 1. The procedure for CMP-ED synthesis was as follows: 1,1,2,2-tetrakis (4-bromophenyl) ethene (648 mg), 1,4-diethynylbenzene (504.6 mg), CuI (66.67 mg), and tetrakis-(triphenylphosphine) palladium (133.33 mg) were added into a three-mouth flat-bottom glass test tube. The degassing operation was performed through vacuuming the tube three times, followed by nitrogen backfill. Then 8 mL toluene and 8 mL triethylamine were added into the tube and the mixture were heated up to 80 °C under magnetic stirring with nitrogen protection. After the solution became uniform mixture, the magneton was removed and the reaction lasted another 72 h. The product was obtained as the temperature cooled down naturally and washed by dichloromethane (3 × 10 mL), acetone (3 × 10 mL), deionized water (3 × 10 mL) and methanol (3 × 10 mL) to remove the remaining monomers and catalyst. Finally, the materials were further purified by Soxhlet extraction for 3 days and treated in vacuum oven at 60 °C for 12 h. The procedure for CMP-PT synthesis was similar to CMP-ED. Only difference was the 1,1,2,2-tetrakis (4-bromophenyl) ethene and 1,4-diethynylbenzene were replaced by 1,3,6,8-tetrabromo-pyrene and 1,3,5-triethynyl-benzene.

## 3 Results and discussion

**Fig. 1:**
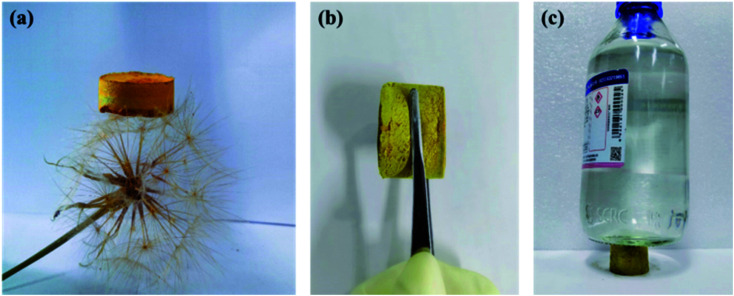
Appearance of CMP-ED foam (a), (b) and compressive resistance test (c).

In Fig. S2,† the X-ray powder diffraction (PXRD) indicated that the CMP-ED and CMP-PT foams were amorphous, with large diffraction peaks of steamed bread in the range of 15–35°. As Fig. 1 showed, two kinds of yellow foams were obtained successfully without physical stirring. The CMP-ED and CMP-PT monolithic foams unveiled ultralight weight and can be propped up by a dandelion (Fig. 1a). The as-synthesized (CMP-ED and CMP-PT) foams exhibited a low apparent density of 58 mg cm^−3^ and 63 mg cm^−3^. Meanwhile, the generated foams exhibited a good mechanical strength in Fig. 1b and load-bearing capacity in Fig. 1c. Therefore, it was seen that the generated foams presented some rigidity after extruding with a compressive strength of up to 0.63 MPa, even if there were reagent bottles filled with 500 g water, the foam was not damaged (Fig. 1b and c).

**Fig. 2:**
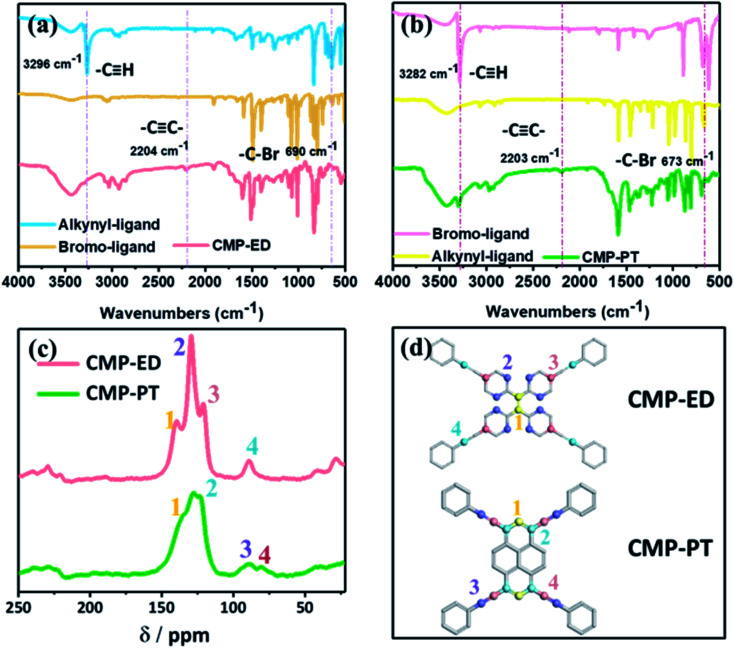
FTIR spectra of monomers and CMP-ED (a) and CMP-PT (b); ^13^C NMR spectra of CMP foams (c); the simulated structure of CMP foams and the label number represented carbon structures corresponding to ^13^C NMR spectra (d).

The CMP structures were firstly characterized by Fourier transform infrared spectroscopy (FTIR) in Fig. 2a and b. The strong absorption peak located near 3450 cm^−1^ was attributed to the stretching vibration of –OH^35^ and aromatics-H (Ar–H) peaks appeared around 3100 to 3000 cm^−1^. The disappearance of the peak around 3300 cm^−1^ belonged to –C≡C–H in ligands and the emergence of the characteristic peak at 2204 cm^−1^ in polymers ascribed to –C≡C– stretching vibration^36^ indicated that the CMP foams were prepared successfully. The peak at 1600 to 1500 cm^−1^ was assigned to benzene skeleton vibration. The peak of =C–H out-of-plane bending vibration was located at 881 to 693 cm^−1^. The peaks at 680 cm^−1^ for CMP-PT and 550 cm^−1^ for CMP-ED were ascribed to –C–Br from the trace unreacted ligands.

The more detailed skeleton information of CMP foams was further validated by ^13^C solid-state nuclear magnetism (NMR) spectra in Fig. 2c, which gave the different local environments around C nuclei and provided detailed structural information. For CMP-ED, resonances of –C=C– units were observed at 140 ppm (the peak 1 which represented carbon 1 of CMP-ED in Fig. 2d) and 89 ppm (the peak 4 which represented carbon 4 of CMP-ED in Fig. 2d) were attributable to –C≡C– units.^37^ The strong signal at 130 ppm (the peak 2 which represented carbon 2 of CMP-ED in Fig. 2d) was assigned to Ar–C which was bonded with hydrogen and the peak at 121 ppm (the peak 3) belonged to the rest carbons of benzene (carbon 3 of CMP-ED in Fig. 2d). CMP-PT contained a similar conjugated framework with CMP-ED. The strong chemical shift signals of Ar–C and –C≡C– units were found at 130 ppm and 90 ppm, respectively. In particular, the feature of the pyrene unit was observed at 135 ppm and 128 ppm. Both structure results proved two CMP foams were synthesized successfully.

**Fig. 3:**
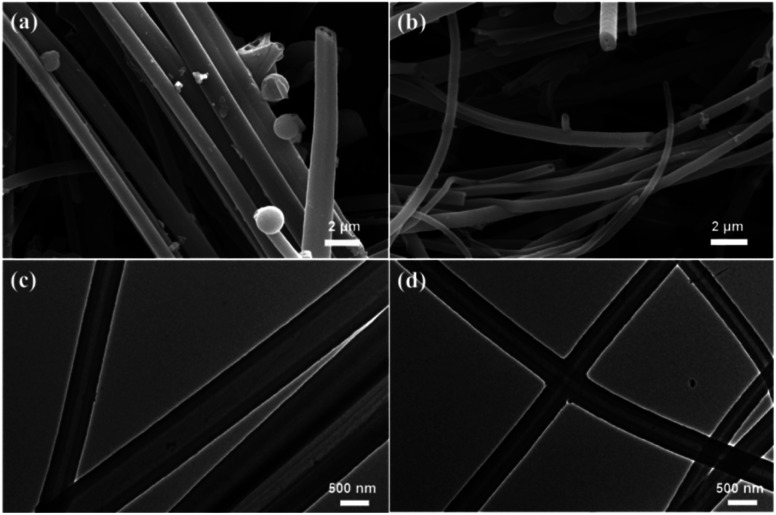
SEM (a) and TEM (c) pictures of CMP-PT, and SEM (b) and TEM (d) pictures of CMP-ED. Scale bar: SEM-2 μm; TEM-500 nm.

The scanning electron microscope (SEM) images were shown in Fig. 3a and b, and indicated that both CMP-ED and CMP-PT possessed open hollow tubes. The cross-section of the two tubes in Fig. 3c and d also demonstrated the hollow tube morphology. The diameter of CMP-ED ranged from 50 nm to 500 nm, while CMP-PT exhibited a larger diameter with a size between 300 nm to 1 μm. For CMP-ED, the tube wall thickness was about 100 nm which was similar to CMP-PT. Therefore, the CMP-ED presented a narrower hollow space due to the smaller diameter compared to CMP-PT.

**Fig. 4:**
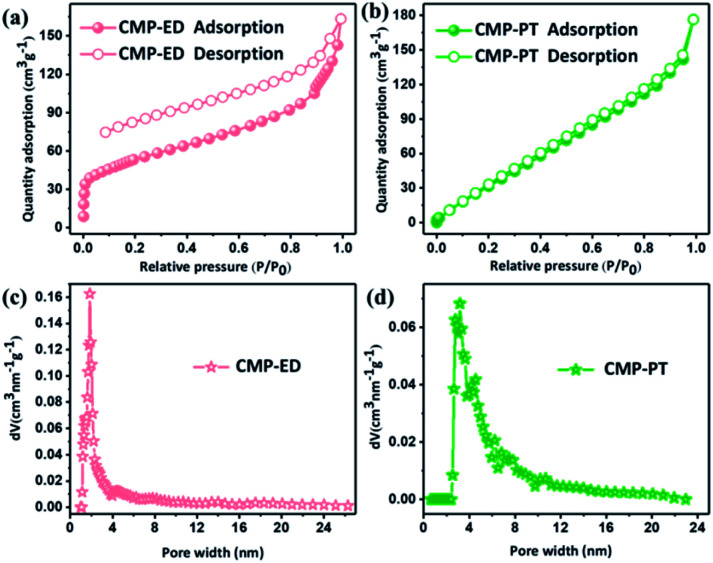
Nitrogen adsorption and desorption isotherms (a) and (b), and distribution of pore size of CMP foams (c) and (d).

The porosity of CMP-ED and CMP-PT were investigated by nitrogen adsorption at 77 K. The nitrogen adsorption and desorption isotherms and pore size distribution were shown in Fig. 4. In Fig. 4a, there was a sharp uptake below *P*/*P*_0_ = 0.05, illustrating the presence of micropores in CMP-ED of type IV isotherm.^38^ The pore size distribution calculated by Density Functional Theory (DFT) method according to adsorption data was concentrated at 1.84 nm for CMP-ED foam, which revealed the rich micro-porous structures and less mesoporous structure. The specific surface area obtained by the DFT method and total pore volumes were 193 m^2^ g^−1^ and 0.2526 cm^3^ g^−1^ at *P*/*P*_0_ = 0.99. It was found that the desorption profile and the adsorption one were not completely closed, revealing that there was still nitrogen molecular left in the framework channel at low pressure for its micro-porous structure. In addition, CMP-PT displayed type II isotherm according to the IUPAC classification. The pore size distribution demonstrated its mesoporous structure with a size between 2.4–4.8 nm. The specific surface area and total pore volumes were 191 m^2^ g^−1^ and 0.2737 cm^3^ g^−1^.

**Fig. 5:**
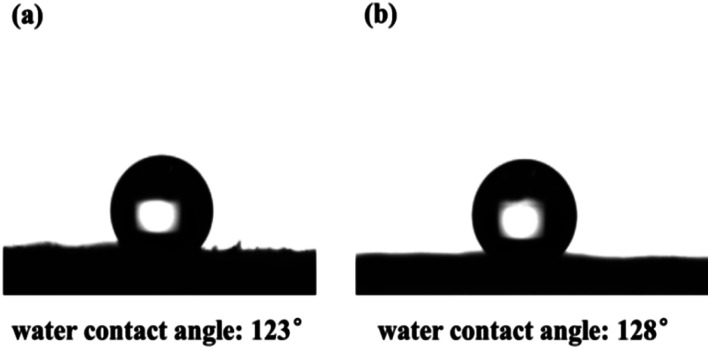
Water contact angle of CMP-PT (a) and CMP-ED (b).

Also, the hydrophobicity of the CMP foams was estimated by contact angle meter in Fig. 5. It was found that both CMP-PT and CMP-ED presented excellent hydrophobicity with a water contact angle of 123° and 128°, respectively. The main reasons leading to the excellent hydrophobicity of materials were the strong hydrophobic aromatic ring and large conjugated structure of gels.

**Fig. 6:**
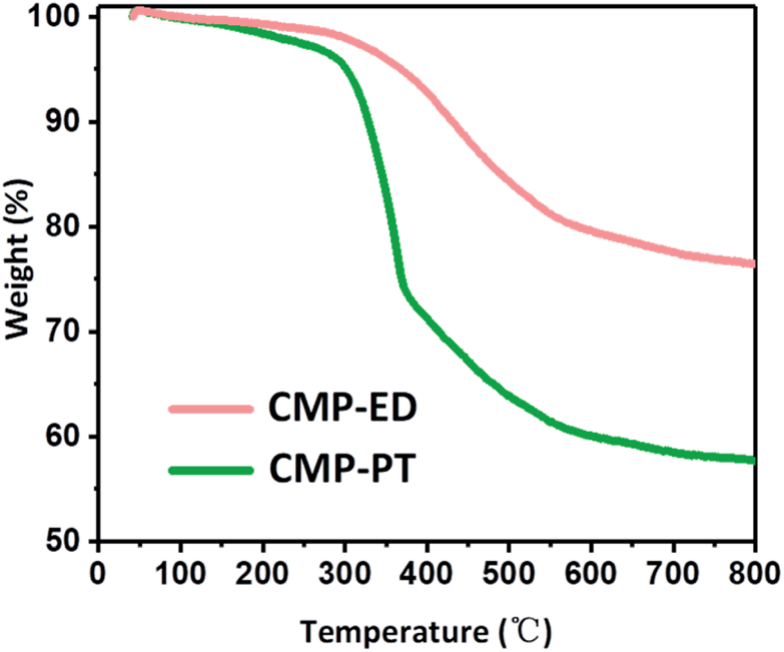
TGA curves of CMP-ED and CMP-PT foams.

Thermal stability of CMP-ED and CMP-PT were investigated by TGA measurement under argon protection in Fig. 6. It was seen CMP-ED lost 25 wt% weight within 50–800 °C, exhibiting excellent thermal stability. The weight loss below 300 °C was attributed to the water and remained ligands evaporation in the frameworks. The obvious inflection point of weight loss around 300 °C was due to the cracking of the polymer in the network. For CMP-PT, the weight loss reached 45% after the test, which was more severe than CMP-ED. The major weight loss of CMP-PT also started around 300 °C and showed a sharp downward trend due to decomposition of the polymer network. In addition, the composition of CMP-PT and CMP-ED before and after TGA was analyzed by an organic element analyzer and ion chromatograph. It was found that the proportion of C element increased significantly after TGA, while H and Br content greatly declined, indicating that the weak bonds such as –C–H and –C–Br bonds were broken during the temperature ramping. (Table S1†). Furthermore, the FTIR spectra of CMP-ED and CMP-PT after TGA showed the characteristic peaks during the structural fingerprint region disappeared, proving the disappearance of the bond during the fingerprint region, such as Ar–H and *et al.* (Fig. S5†).

**Fig. 7:**
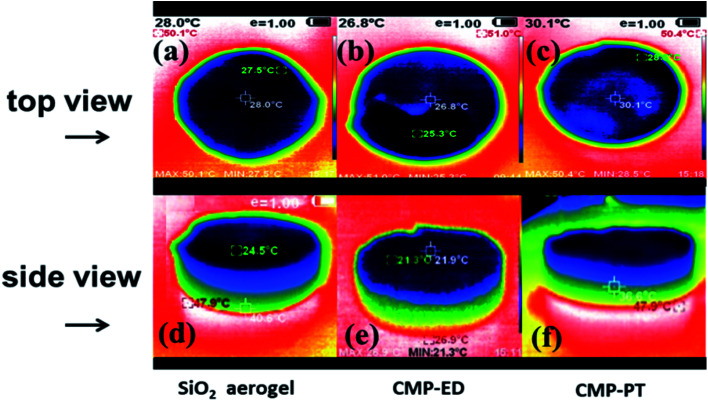
Thermographic images of (a and d) SiO_2_ aerogel, (b and e) CMP-ED, and (c and f) CMP-PT at 323.15 K. In the images, the red area was the heating table, and the boundary between the red and yellow-green area was the contact surface between the foam block and the heating table.

**Fig. 8:**
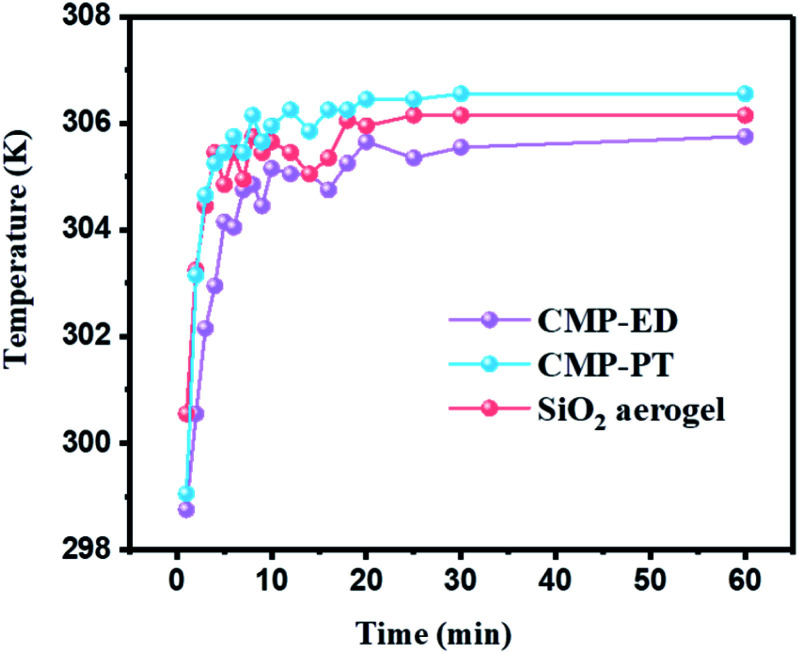
Surface temperature variation curves of CMP-ED, CMP-PT, and SiO_2_ aerogel as a function of running time under 323.15 K condition.

**Fig. 9:**
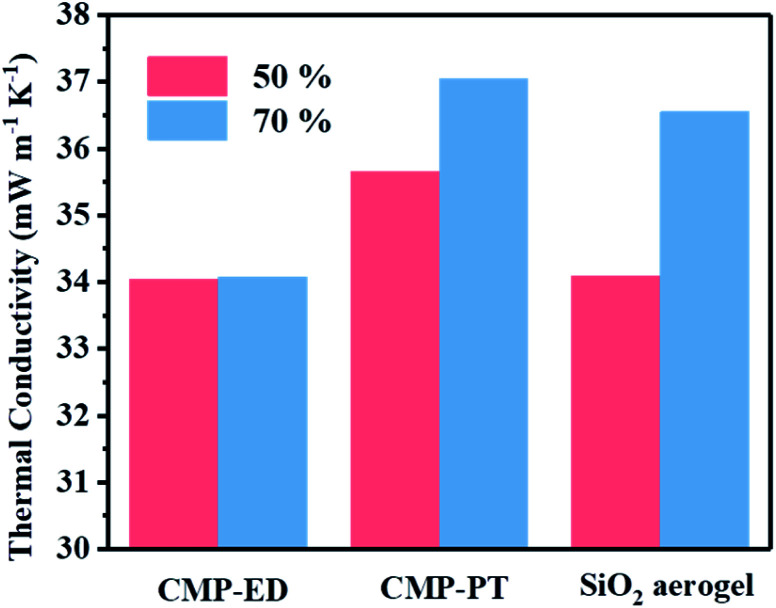
Thermal conductivity data comparation of CMP-ED, CMP-PT and commercial SiO_2_ aerogel in 50% humidity (pink) and 70% humidity (blue) environment.

To study the thermal insulation performance of the CMP foams, the temperature distribution from the bottom to the surface during the heating process was recorded by the infrared thermal imager. The images shot from a top view (a–c) and side view (d–f) were shown in Fig. 7. When the temperature of the heating table reached 323.15 K, the temperature at the top of the material was 299.95 K for CMP-ED. Compared with SiO_2_ aerogel, CMP-ED presented the lowest temperature from the different angles, while CMP-PT showed the highest temperature among them. We also studied the thermal insulation performance of the CMP foams at different temperatures, and the results showed that these foams were effective in trapping heat at the bottom consistently (Fig. S6 and S7†). In Fig. 8, the surface temperature variation of the foams was studied under 323.15 K condition to reveal their heat insulation performance as a function of running time. For three materials, CMP-ED took the longest time to achieve an equilibrium temperature, proving its strongest ability to inhibit heat transfer. Meanwhile, the equilibrium temperature of CMP-PT was highest, while the temperature of CMP-ED was the lowest, illustrating that CMP-ED foam performed the best heat insulation performance for its lowest thermal conductivity compared with SiO_2_ aerogel and CMP-PT. To investigate the thermal insulation performance of CMP foams in humid environments, the thermal conductivity of the foams was measured at 50% and 70% humidity referring to commercial SiO_2_ aerogels. The results showed in Fig. 9 and Table S2† revealed that CMP-ED foam exhibited comparable thermal insulation conductivity with the commercial SiO_2_ aerogel at 50% humidity, while CMP-PT foam presented higher thermal conductivity. The thermal conductivity values of the CMP foams were 34.04 mW m^−1^ K^−1^ for CMP-ED and 35.65 mW m^−1^ K^−1^ for CMP-PT, respectively, and 34.09 mW m^−1^ K^−1^ for commercial SiO_2_ aerogels. However, with the increase of humidity to 70%, CMP foams and SiO_2_ aerogel exhibited different thermal insulation properties. The thermal conductivity of CMP-ED and CMP-PT increased by 0.12% and 3.93%, respectively, while the commercial SiO_2_ aerogels increased by as much as 7.22%. The thermal conductivity attenuation coefficient of SiO_2_ aerogels was 60 times as much as CMP-ED. It can be seen that CMP foams maintained excellent thermal insulation performance at high humidity, which was possible to be a candidate for excellent thermal isolation materials in high humidity environments such as the freshwater lake. Furthermore, we compared the thermal conductivity of CMP foams with other polymers reported previously (Fig. S8†). For example, the phenolic foam CCIM-126 showed a thermal conductivity of 50 mW m^−1^ K^−1^, which was much higher than CMP-ED and CMP-PT. Compared with those reported polymers, the thermal insulation advantages of CMP foams were still significant.^39^

Based on the bulk characterization results, it was found the pore structure and the hydrophobicity of materials affected their thermal conductivity. Firstly, according to the BET measurement (Fig. S3†), the specific surface area of SiO_2_ aerogel was 79 cm^3^ g^−1^, and it presented a mesoporous structure with a pore size beyond 2 nm. The pore size of CMP-ED was smaller than commercial SiO_2_ aerogel which possessed a micro-porous structure. The narrow pore structure of CMP-ED inhibited the gas diffusion and the consequent heat transfer instinctively. Furthermore, it was known the pore structure of SiO_2_ aerogel generated from the piling up of SiO_2_ particles, compared with the long and narrow pore structure of CMP-ED in Fig. 3, CMP-ED needed higher driving force to promote the gas diffusion. Additionally, the hydrophobicity of CMP-ED made the foams avoid being soaked. At room temperature and pressure, the thermal conductivity of liquid water (about 590 mW m^−1^ K^−1^) was much higher than that of air (about 26 mW m^−1^ K^−1^). Compared with the SiO_2_ aerogel, the thermal insulation performance of CMP-ED was mostly not affected by the moisture in humid conditions. And both factors contributed to the low and impervious thermal conductivity of CMP-ED. In addition, the water contact angle of aerogel SiO_2_ was 104.5° in Fig. S4.† Compared with CMP-ED, CMP-PT possessed a comparable hydrophobicity property, but its bigger pore size and micro-porous structure induced higher thermal conductivity. Compared with CMP-ED, CMP-PT and SiO_2_ aerogel presented less hydrophobic, which could introduce more water molecules into the material framework, enhancing heat transfer and weakening their heat insulation effect. Besides, from the pore size distribution results, the CMP-ED foam mainly showed 1.84 nm micropores, while CMP-PT and SiO_2_ aerogel possessed larger pore sizes. It was deduced that the smaller pore size inhibited air diffusion and the consequent heat transfer, determining their heat insulation performance and explaining the higher thermal conductivity of CMP-PT and SiO_2_ aerogel and lower one for CMP-ED.

## 4 Conclusions

In summary, two CMP foams with low density, superhydrophobic, excellent physicochemical stability, and low thermal conductivity have been synthesized. Through measuring the thermal conductivity of the CMP foams under different humidity conditions, it was proved that the thermal conductivity of CMP-ED material changed only 0.12% when humidity increased to 70% from 50%, which presented much better insulation performance than commercial SiO_2_ aerogels. The bulk characterization results demonstrated that the strong hydrophobicity of CMPs weakened the gas diffusion and water vapor movement internally and externally in humid conditions. Besides, the narrow aperture limited the airflow and inhibited the consequent heat transfer. Both of them enhanced the insulation properties of the foams under humid conditions. It can be seen that CMP foams maintained excellent thermal insulation performance and presented a great potential to be an insulation material in high humidity environments such as the freshwater lake. More importantly, this work gave more tips for insulation CMP foams design and synthesis in the future.

## Conflicts of interest

There are no conflicts to declare.

## Supplementary Material

RA-011-D1RA01616D-s001
