# Supplementary material for: Conjugated microporous polymer foams with excellent thermal insulation performance in a humid environment
Source: RSC Adv. 2021 Apr 14;11(23):13957–63. doi: 10.1039/d1ra01616d (PMC8697720; doi:10.1039/d1ra01616d)
Supplement: RA-011-D1RA01616D-s001 [file RA-011-D1RA01616D-s001.pdf]

Electronic Supplementary Material (ESI) for RSC Advances.
This journal is © The Royal Society of Chemistry 2021

Supporting Information

Conjugated microporous polymer foams with excellent thermal insulation performance in humid environment

Experiment materials

1,1,2,2-tetrakis(4-bromophenyl) ethene (Kylpharm Co. Ltd), 1,3,6,8-tetrabromo-pyrene (Kylpharm Co. Ltd), 1,4-diethynylbenzene (Kylpharm Co. Ltd), 1,3,5-triethynyl-benzene (Kylpharm Co. Ltd), copper(I) iodide, tetrakis (triphenylphosphine) palladium, toluene, triethylamine.

Characterization

The structure of CMP foams was characterized by Solid-state ^{13}C-CP/MAS NMR spectroscopy and Fourier Transform Infrared Spectroscopy (FTIR). Nitrogen isotherms were measured at 77K by Autosorb-iQ automatic specific surface and pore size distribution analyzer, Quanta-chrome. Powder X-ray diffraction (PXRD) was investigated on a D-MAX X-ray diffractometer (Rigaku, Japan). Scanning electron microscopy (SEM) images were operated on Apreo S instrument, (FEI American). Transmission electron microscopy（TEM）images were performed on TECNAI G^2 TF20 at an accelerating voltage of 200 kV, (FEI American). The powder was dispersed in EtOH solution and a drop mixture was dropped onto the copper network. TGA curves were obtained under argon protection by STA 449 F5 (NETZSCH), and the temperature was elevated from 50℃ to 800℃ with a heating rate of 10℃ min^{-1}. DSA100 was used in measuring the water contact angle, (KRUSS, Germany).

Test of thermal conductivity

Multifunction-rapid thermal conductivity tester（DRE-Ⅲ, Xiang-yi）was used in measurement of CMP foams under different humidity conditions(50%/70%). Reference standard is ISO22007-2.

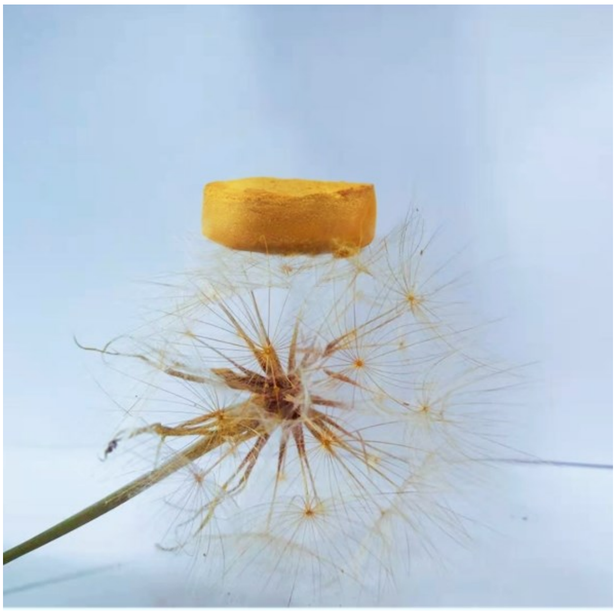

Figure S1 The image of CMP-PT foam

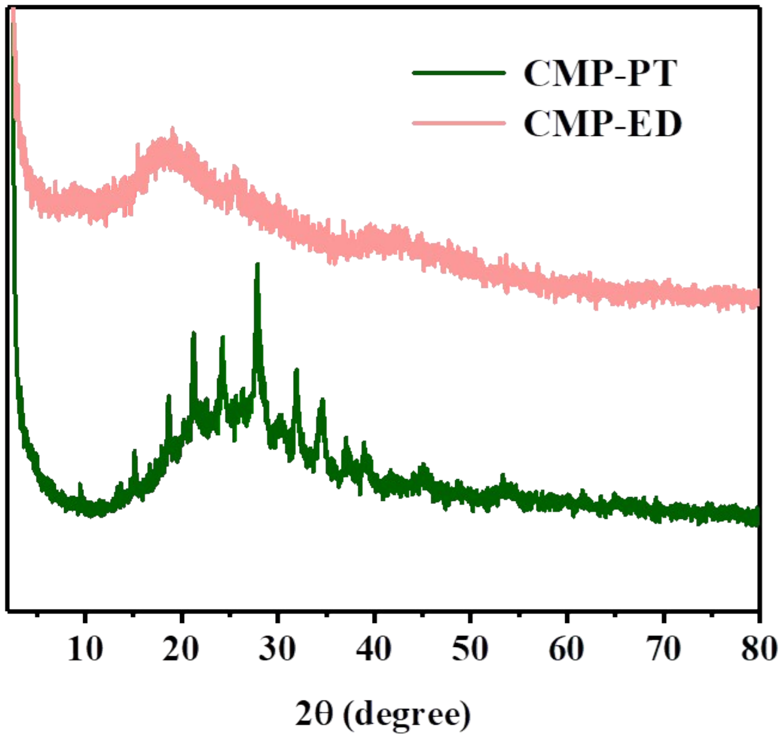

Figure S2 Powder X-ray diffraction patterns of CMP-ED and CMP-PT

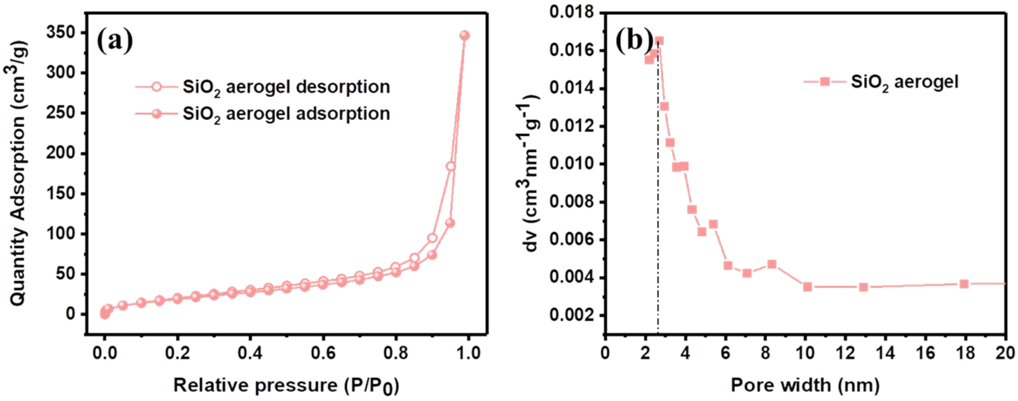

Figure S3 Nitrogen adsorption and desorption isotherms (a), and distribution of pore size (b) of SiO_2 aerogel.

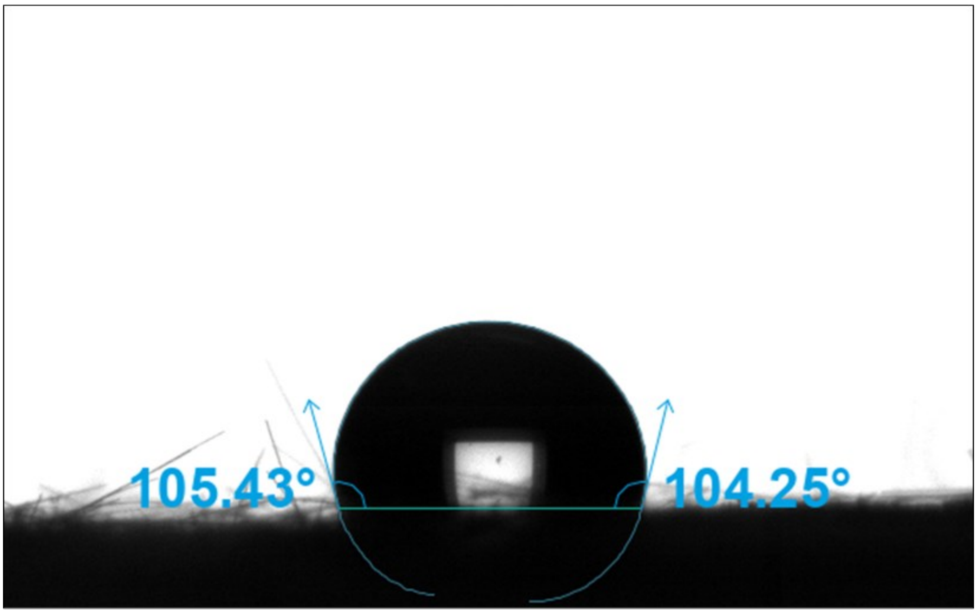

Figure S4 Water contact angle of SiO_2 aerogel

Table S1 The elemental analysis of CMP-ED and CMP-PT

Samples	The ratio of content of H: C (mol: mol)	The ratio of content of Br: C (mol: mol)
CMP-ED	69.13:100	1.73:100
CMP-PT	61.30:100	6.49:100
CMP-ED after TGA	11.07:100	0
CMP-PT after TGA	9.1:100	0

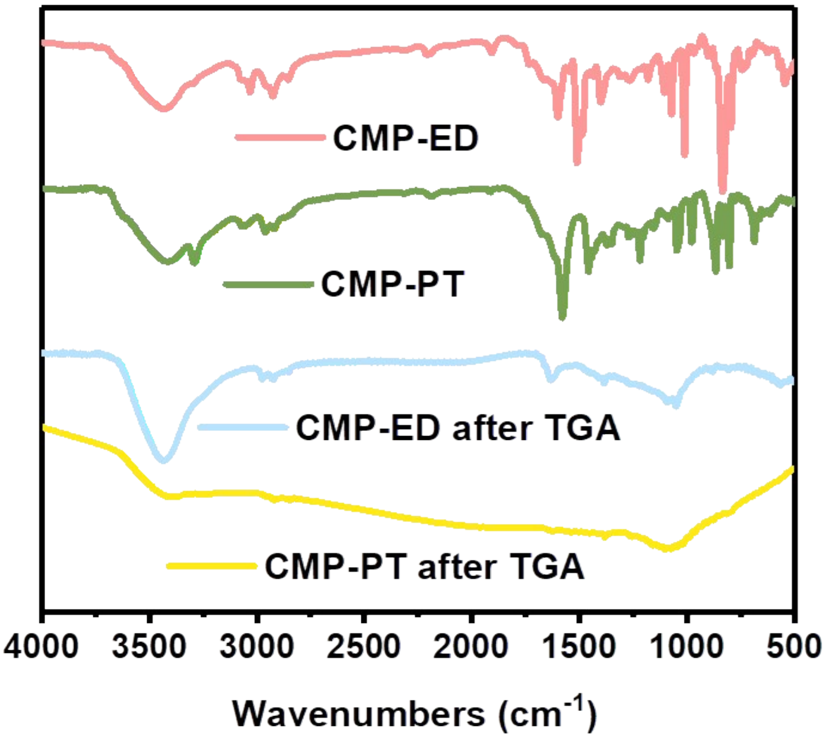

Figure S5 the FTIR spectra of CMP-PT and CMP-ED before and after TGA

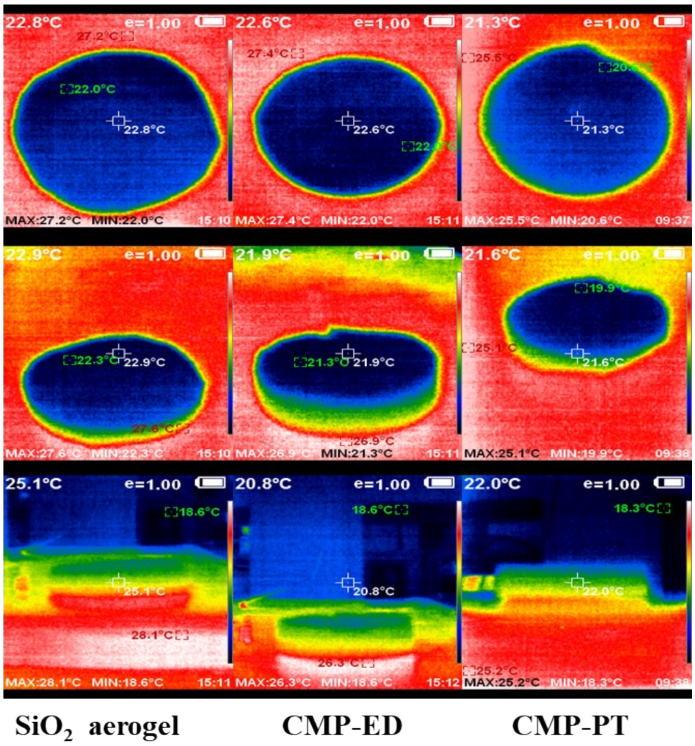

Figure S6 Thermographic images of SiO_2 aerogel, CMP-ED and CMP-PT at 298.15 K

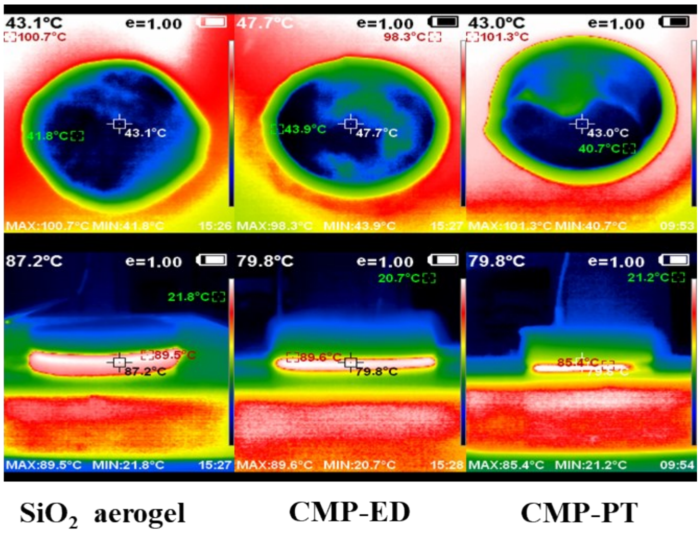

Figure S7 Thermographic images of SiO_2 aerogel, CMP-ED and CMP-PT at 398.15 K

Table S2 Thermal conductivity datas of CMP-ED, CMP-PT and commercial SiO_2 gel

Test environmental humidity	Thermal conductivity （W $m^{-1}K^{-1}$)		
	CMP-ED	CMP-PT	commercial SiO_2 gel
50%	0.03404	0.03565	0.03409
70%	0.03408	0.03705	0.03655
Value changed (%)	0.12	3.93	7.22

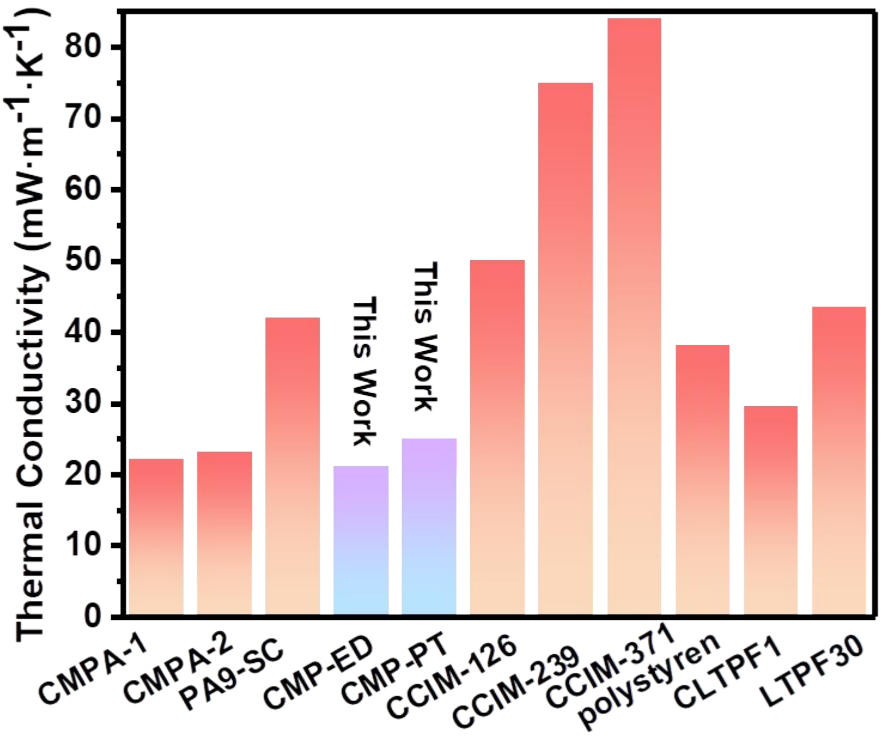

Figure S8 Comparison of thermal conductivity between CMP foams and other polymers [1-3]

Reference

1. J. Li, A. Zhang, S. Zhang, Q. Gao, W. Zhang and J. Li, *Composites Part B: Engineering*, 2019, **156**, 368-377.
2. L. Wang, Y.-K. Wu, F.-F. Ai, J. Fan, Z.-P. Xia and Y. Liu, *Polymers*, 2018, **10**, 1310.
3. P. Mu, W. Bai, Z. Zhang, J. He, H. Sun, Z. Zhu, W. Liang and A. Li, *Journal of Materials Chemistry A*, 2018, **6**, 18183-18190.
